# Correlation of SARS-CoV-2-breakthrough infections to time-from-vaccine

**DOI:** 10.1038/s41467-021-26672-3

**Published:** 2021-11-04

**Authors:** Barak Mizrahi, Roni Lotan, Nir Kalkstein, Asaf Peretz, Galit Perez, Amir Ben-Tov, Gabriel Chodick, Sivan Gazit, Tal Patalon

**Affiliations:** 1 KI Research Institute, Kfar Malal, 4592000 Israel; 2 Kahn Sagol Maccabi (KSM) Research & Innovation Center, Maccabi Healthcare Services, Tel Aviv, 68125 Israel; 3 Internal Medicine COVID-19 Ward, Samson Assuta Ashdod University Hospital, Ashdod, Israel; 4 Sackler Faculty of Medicine, Tel Aviv University, Tel Aviv, Israel; 5 Maccabitech Institute for Research and Innovation, Maccabi Healthcare Services, Tel Aviv, Israel

**Keywords:** RNA vaccines, SARS-CoV-2, Epidemiology

## Abstract

The short-term effectiveness of a two-dose regimen of the BioNTech/Pfizer mRNA BNT162b2 severe acute respiratory syndrome coronavirus 2 (SARS-CoV-2) vaccine was widely demonstrated. However, long term effectiveness is still unknown. Leveraging the centralized computerized database of Maccabi Healthcare Services (MHS), we assessed the correlation between time-from-vaccine and incidence of breakthrough infection between June 1 and July 27, the date of analysis. After controlling for potential confounders as age and comorbidities, we found a significant 1.51 fold (95% CI, 1.38–1.66) increased risk for infection for early vaccinees compared to those vaccinated later that was similar across all ages groups. The increased risk reached 2.26- fold (95% CI, 1.80–3.01) when comparing those who were vaccinated in January to those vaccinated in April. This preliminary finding of vaccine waning as a factor of time from vaccince should prompt further investigations into long-term protection against different strains.

## Abstract (web summary)

The duration of effectiveness of SARS-CoV-2 vaccination is not yet known. Here, the authors present preliminary evidence of BNT162b2 vaccine waning across all age groups above 16, with a higher incidence of infection in people who received their second dose early in 2021 compared to later in the year.

## Introduction

A two-dose regimen of the BioNTech/Pfizer mRNA BNT162b2 severe acute respiratory syndrome coronavirus 2 (SARS-CoV-2) vaccine was demonstrated to be highly effective in preventing infection and symptomatic COVID-19, both in clinical trials^1,2^ and real-world settings^3,4^. However, long-term effectiveness is still unknown. Different studies examined the immunological response over time^5–7^ whereas other smaller cohort studies have analyzed serological response in relation to clinical outcomes^8^, large-scale studies of a long-term correlate of protection are still needed.

In addition, differentiating between time-from-vaccine and vaccine effectiveness across different strains^9,10^ is challenging, as new Variants of Concern are rapidly identified.

The Delta (B.1.617.2) variant, initially identified in India and now globally detected, is currently the dominant strain in Israel. In light of the recent surge of cases in Israel, many of which among vaccinated individuals^11^, concerns of reduced vaccine efficacy against the Delta variant have surfaced, including official reports of decreased protection (https://www.gov.il/he/Departments/news/05072021-03). Contrastingly, other studies report only modest differences in vaccine effectiveness^12^ and substantial antibody response to the Delta variant^13^.

Data regarding the duration of protection are essential for effective resource allocation and vaccine administration, such as the need and urgency of a third dose^14,15^. Israel’s rapid rollout of the mass vaccination campaign allows us to investigate the correlation between time-from-vaccine and vaccine effectiveness against the Delta variant.

To this end, we conducted a retrospective cohort study comparing the incidence rates of breakthrough infections and COVID-19-related hospitalization between Early and Late Vaccinees, using data from Maccabi Healthcare Services (MHS), Israel’s second-largest Health Maintenance Organization, which covers 2.5 million members (26.5% of the population) and provides a representative sample of the Israeli population.

## Results

Of 1,395,134 MHS members over the age of 16 who received the second dose of the vaccine between January and April of 2021, 1,352,444 were eligible for the study. In all, 27,143 individuals did not receive the second dose according to the guidelines, and 15,547 individuals were tested positive for SARS-CoV-2 prior to the study period.

**Fig. 1:**
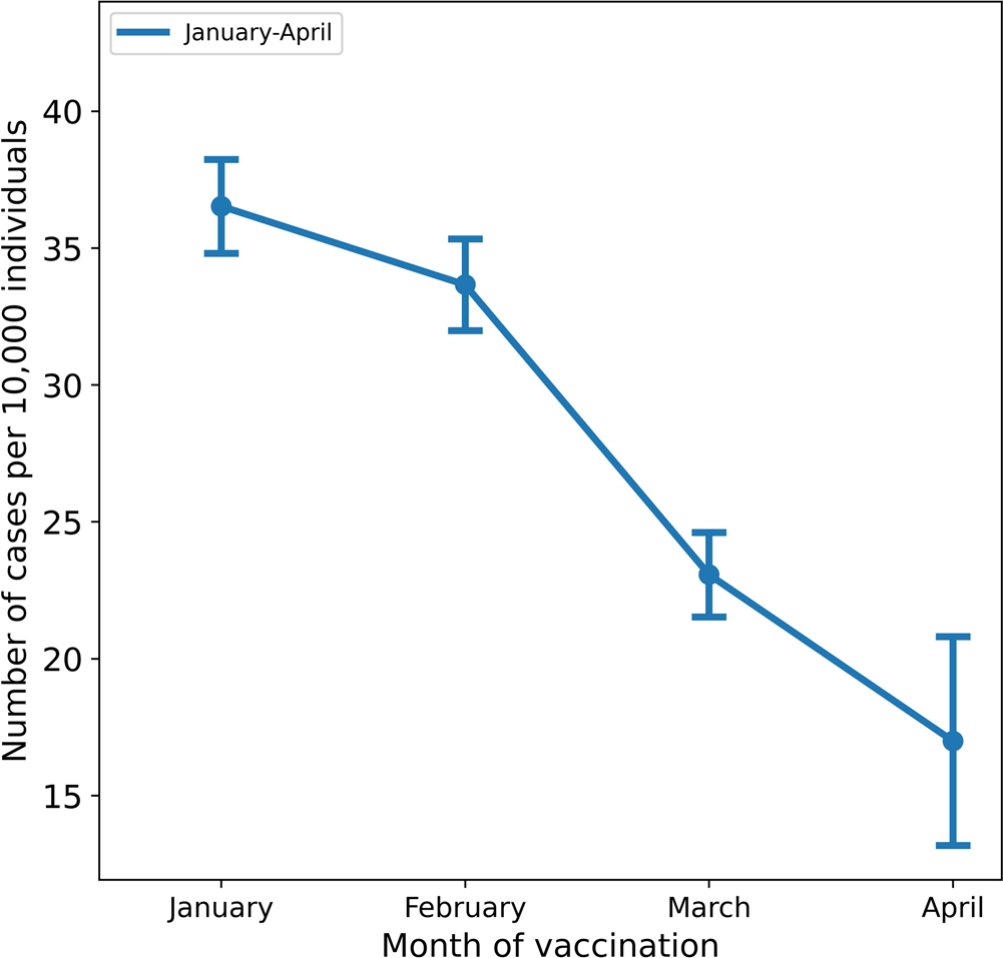
Crude breakthrough infections by month of vaccination. The crude incidence rates per 10,000 individuals above the age of 16 are shown by the month of administration of the second dose of the vaccine; bars represent 95% confidence intervals.

The incidence rates of breakthrough infections per 10,000 individuals who received their second dose during the months of January, February, March, and April were 36.5 (95% CI 34.8–38.2), 33.65 (95% CI 31.9–35.3), 23.06 (95% CI 21.5–24.6), and 16.98 (95% CI 13.1–20.8), respectively (Fig. 1).

**Fig. 2:**
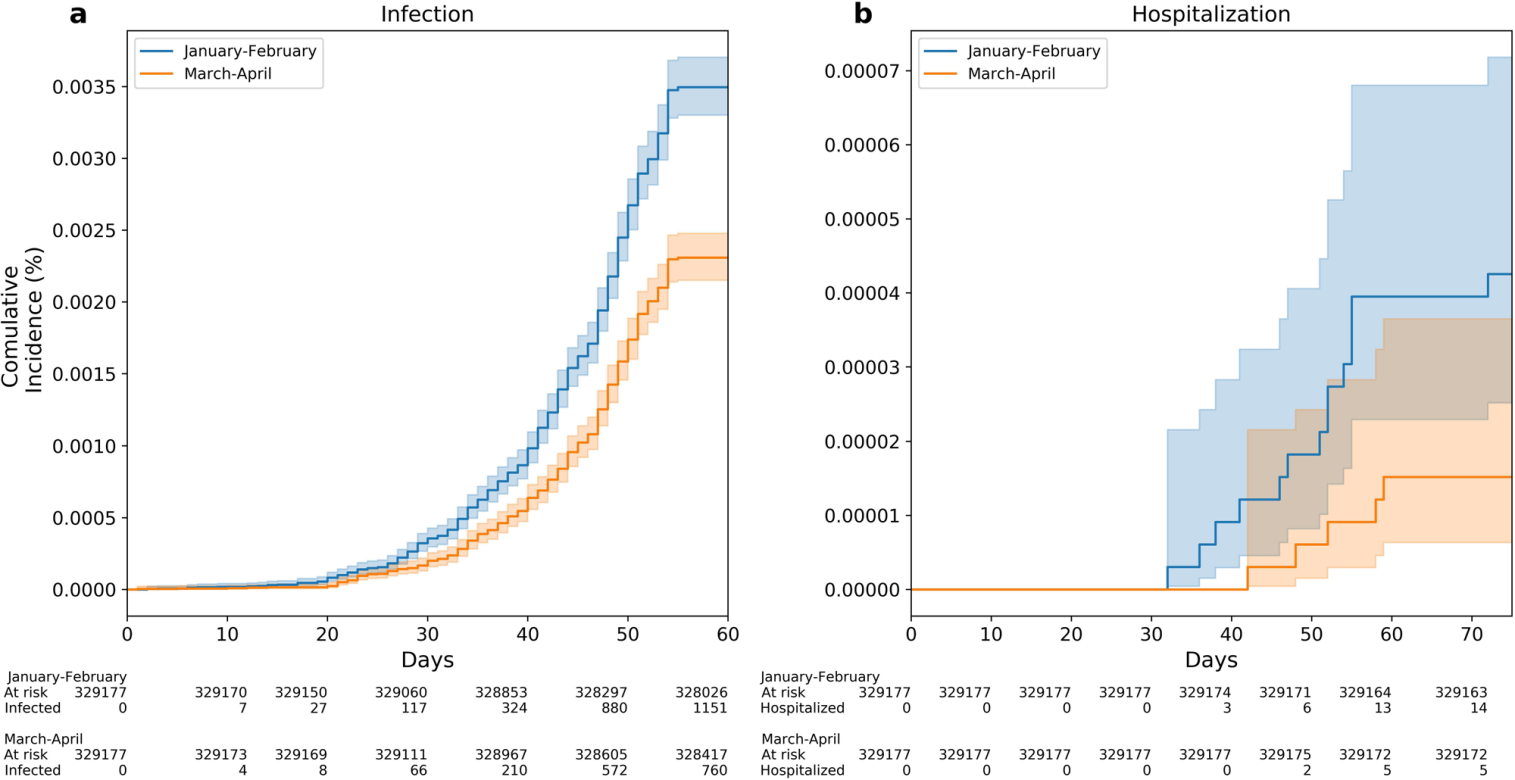
Accumulative breakthorough infections and hospitalizations among Early and Late Vaccinnes during June - July 2021, Israel. Kaplan–Meier curves are used for the accumulative probability of breakthrough infection (**a**) and hospitalization (**b**) between June and July 2021 among individuals who were vaccinated Early (January to February 2021) compared with individuals who were vaccinated late (March to April 2021). *X* axis in curves **a**, **b** represents days from the start of the follow-up period (June 1, 2021). Shading illustrates 95% CIs (**a**, **b**).

In the first model, we compared the incidence rate among individuals who were vaccinated during January and February (Early Vaccinees group) vs. those who were vaccinated during March and April (Late Vaccinees group). We matched 329,177 individuals in each group based on age group (18–39, 40–59, and 60 and over), sex, city of residence, and socioeconomic status (SES). During the follow-up period (between June 1 and July 27), 1911 cases of breakthrough infection were recorded, 1151 of them in the Early Vaccinees group and 760 in the Late Vaccinees group. After adjustment for underlying comorbidities, we found a statistically significant 51% (95% CI 40–68%) increased risk for breakthrough infection in Early Vaccinees (*P* < 0.001) (Table 1; Model 1, Fig. 2). When stratifying the results by age, we found a similar trend across all age groups. In addition, there was a non-significant trend of increased hospitalizations with 15 and 4 in the Early and Late Vaccine groups, respectively (HR = 2.4, 95% CI 0.8–6.7) (Fig. 2). In the second model, we compared each month separately by including individuals according to the month in which they considered fully vaccinated and found higher risks for breakthrough infections in those who were vaccinated Early compared with late in each month-group (Table 1, Model 2). Individuals who were vaccinated in January 2021 had a 2.26-fold increased risk (95% CI 1.80–3.01) for breakthrough infection compared to individuals who were vaccinated in April 2021 (Table 1).

**Table 1:** Hazard ratio (HRs) of SARS-CoV-2-breakthrough infections between Early and Late Vaccinee groups.

	*N* in the Early Vaccinees group	*N* in the Late Vaccinees group	*N* in each matched^a^ group	*N* of new cases^b^ Early	*N* of new cases^b^ Late	HR^c^ (95% CI)	*P*
*Model 1: comparing Early Vaccinees* *(January–February 2021) to Late Vaccinees (March–April 2021)*							
All	935,781	416,663	329,177	1151	760	1.51 (1.38-1.66)	1.51E-18
16–39 yr	242,943	302,229	215,919	807	530	1.52 (1.36-1.69)	1.16E-05
40–59 yr	394,896	86,559	85,502	283	191	1.47 (1.22-1.76)	5.13E-05
≥60 yr	297,942	27,875	27,756	61	39	1.55 (1.03-2.32)	0.035
*Model 2: comparing vaccinees by month in 2021*							
Jan/Feb	475,281	460,500	233,622	1019	795	1.31 (1.20-1.45)	1.43E-08
Jan/Mar	475,281	371,929	145,431	563	351	1.61 (1.41-1.85)	3.68E-12
Jan/Apr	475,281	44,734	40,356	156	70	2.26 (1.70-3.01)	2.24E-08
Feb/Mar	460,500	371,929	272,243	929	666	1.38 (1.25-1.53)	1.48E-10
Feb/Apr	460,500	44,734	43,649	148	75	2.00 (1.51-2.64)	1.14E-06
Mar/Apr	371,929	44,734	44,402	101	76	1.34 (1.00–1.81)	0.052

Table 1 Cox proportional hazards regression models were applied to measure the risk of breakthrough infection depending on time from vaccine in two models.

Explanations and abbreviation: *Jan* January 2021, *Feb* February 2021, *Mar* March 2021, *Apr* April 2021. *P* two-sided *p* value.

^a^Number in each matched group in a 1:1 ratio after matching for sex, age group, socioeconomic status, and city of residence.

^b^New breakthrough infections in the matched groups.

^c^Adjusted for number of PCR tests and underlying comorbidities including obesity, cardiovascular disease, diabetes, hypertension, chronic kidney disease, cancer, COPD, IBD, and immunosuppression conditions.

## Discussion

In this cohort of MHS members, all of whom are vaccinated with the BioNTech/Pfizer mRNA BNT162b2 vaccine in a two-dose regimen, we identified a significant correlation between time-from-vaccine and afforded protection against SARS-CoV-2 infection. The risk for breakthrough infection was significantly higher for Early Vaccinees compared with those vaccinated later with an additional trend for higher risk for hospitalization among the Early Vaccinees group. Our results correspond to recent publications that demonstrate a significant decline in antibody levels and immune systems compounds over time following the second dose of vaccination^5–7^.

Our study has several important limitations. First, as the Delta variant was the dominant strain in Israel during the study period, the observed decrease in long-term protection afforded by the vaccine against other strains cannot be inferred. Second, as we excluded participants with previous infections and the longer time intervals between the two doses, our study cannot assess the effectiveness of these two factors on breakthrough infection and the results may not be generalizable to settings where an extended dosing interval was applied. At last, the results might be affected by differences between the groups in terms of health behaviors (such as social distancing and mask-wearing), a possible confounder that was not assessed. Our adjustment of PCR test frequency mitigated some of the potential bias of testing behavior. As chronically ill patients were given priority for vaccination, confounding by indication should be considered when interpreting the study results; nonetheless, adjusting for obesity, cardiovascular disease, diabetes, hypertension, chronic kidney disease, cancer, chronic obstructive pulmonary disease (COPD), inflammatory bowel disease (IBD), and immunosuppression had only a small impact on the estimate of effect as compared with the unadjusted HR (unadjusted results are provided in Supplementary Table 1). Therefore, residual confounding by unmeasured factors is unlikely.

Taken together, the study suggests a possible relative decrease in the long-term protection of the BNT162b2 vaccine against the Delta variant of SARS-CoV-2. This preliminary finding should be evaluated in future studies, including a comparison to long-term protection against different strains, and prospective clinical trials to examine the effect of a booster vaccine against breakthrough infection.

## Methods

The study population consisted of all MHS members aged 16 and above who received the second dose of the vaccine between January and April 2021. Individuals were considered fully vaccinated if they received two doses of the BNT162b2, the second one administered within the 21-to-28-day interval set by national guidelines. The minority who did not follow the guidelines included those infected after the first dose or those suffering an intercurrent illness that delayed the administration of the second dose.

Individuals were excluded from the study if they had a positive SARS-CoV-2 polymerase chain reaction (PCR) assay test result prior to the start of the study period or disengaged from MHS for any reason between January and April. Individual-level data of the study population included age, sex, city of residence, last documented body mass index (Kg/m^2^) (categorized as normal weight <25, overweight 25–30, and obese >30), and SES, on a scale from 1 (lowest) to 10. SES index is based on several parameters including household income, educational qualifications, household crowding, material conditions, and car ownership. Data collected also included information of chronic diseases from MHS’ automated registries, including cardiovascular diseases^16^, hypertension, diabetes^17^, chronic kidney disease^18^, COPD, IBD, and immunocompromised conditions, as well as data on cancer from the National Cancer Registry^19^. In addition, dates of the first and second dose of the vaccine (if received), count, and results of any PCR tests for SARS-CoV-2, all recorded centrally in MHS, were included in the analysis.

To assess the correlation between time-from-vaccine and afforded protection against breakthrough infection, two Cox proportional hazards regression models were applied. In both models, the outcome was defined as a positive SARS-CoV-2 PCR test recorded between June 1st and July 27th, the date of analysis.

In the first model, we addressed the time-from-vaccine by grouping individuals into two separate groups of comparison: Early Vaccinees and Late Vaccinees. We defined Early Vaccinees as individuals who received the second dose of the vaccine between January and February 2021 and Late Vaccinees as individuals who received the second dose between March and April 2021. As the mass vaccination campaign first targeted high-risk individuals (e.g., healthcare personnel and persons with comorbidities) and those over the age of 60, we matched each Early Vaccinee to a Late Vaccinee individual in a 1:1 ratio, based on age group (18–39, 40–59, and 60 and over), sex, city of residence, and socioeconomic status. Results were then adjusted for underlying comorbidities, including obesity, cardiovascular diseases, diabetes, hypertension, chronic kidney disease, cancer, COPD, IBD, and immunosuppression conditions. In addition, the number of PCR tests each individual performed from the beginning of the pandemic until the beginning of the follow-up period was used as a proxy for COVID-19-related health-seeking behavior by adjusting to it as a categorical variable.

In the first model, we additionally applied Cox proportional hazards regression to calculate the Hazard ratio COVID-19-related hospitalizations between the groups.

In the second model, we addressed the time-from-vaccine by analyzing six distinct groups, comparing individuals according to the month in which they were first considered to be fully vaccinated (the groups were January–February, January–March, January–April, February–March, February–April, and March–April). Thereby, we compared the incidence of SARS-CoV-2-breakthrough infection between individuals who were fully vaccinated in January 2021 and those who were fully vaccinated in February 2021 and so on. The same matching and adjustment methods were performed in both models.

Analyses were performed using Python version 3.1 with the stats model package.

### Reporting summary

Further information on research design is available in the Nature Research Reporting Summary linked to this article.

## Supplementary information

Supplementary Information

Peer Review File

Reporting Summary

## Data Availability

According to the regulations of the Israel Ministry of Health, individual-level data cannot be shared openly. Specific requests for remote access to de-identified community-level data should be referred to Maccabitech, Maccabi Healthcare Services Institute for Research & Innovation.

## Code availability

Analysis code is available at https://github.com/barakm-ki/Correlation-of-SARS-CoV-2-Breakthrough-Infections-to-Time-from-vaccine.

## References

[1] Polack FP (2020). Safety and efficacy of the BNT162b2 mRNA covid-19 vaccine. N. Engl. J. Med..

[2] Baden, L. R. et al. Efficacy and Safety of the mRNA-1273 SARS-CoV-2 vaccine. *N. Engl. J. Med.* **384**, 403–416 (2020). 10.1056/NEJMoa2035389 PMC7787219 33378609

[3] Dagan, N. et al. BNT162b2 mRNA covid-19 vaccine in a nationwide mass vaccination setting. *N. Engl. J. Med.* **384**, 1968-1970 (2021). 10.1056/NEJMc2104281 33882227

[4] Chodick, G. et al. The effectiveness of the two-dose BNT162b2 vaccine: analysis of real-world data. *Clin. Infect. Dis.*10.1093/CID/CIAB438 (2021). 10.1093/cid/ciab438 PMC8240867 33999127

[5] Seow J (2020). Longitudinal observation and decline of neutralizing antibody responses in the three months following SARS-CoV-2 infection in humans. Nat. Microbiol..

[6] Ruopp MD, Strymish J, Dryjowicz-Burek J, Creedon K, Gupta K (2021). Durability of SARS-CoV-2 IgG antibody among residents in a long-term care community. J. Am. Med. Dir. Assoc..

[7] Shrotri, M. et al. Spike-antibody waning after second dose of BNT162b2 or ChAdOx1. *Lancet* **398**, 385-387 (2021). 10.1016/S0140-6736(21)01642-1 PMC8285117 34274038

[8] Bergwerk, M. et al. Covid-19 breakthrough infections in vaccinated health care workers. *N. Engl. J. Med.*10.1056/NEJMoa2109072 (2021). 10.1056/NEJMoa2109072 PMC8362591 34320281

[9] Liu, Y. et al. BNT162b2-elicited neutralization against new SARS-CoV-2 spike variants. *N. Engl. J. Med.*10.1056/NEJMc2106083 (2021). 10.1056/NEJMc2106083 PMC8133696 33979486

[10] Wang, P. et al. Increased resistance of SARS-CoV-2 variant P.1 to antibody neutralization. *bioRxiv*, 10.1101/2021.03.01.433466 (2021). 10.1016/j.chom.2021.04.007 PMC8053237 33887205

[11] COVID-19 in Israel dashboard. (2021).

[12] Bernal, J. L. et al. Effectiveness of Covid-19 vaccines against the B.1.617.2 (Delta) variant. *N. Engl. J. Med.*10.1056/NEJMoa2108891 (2021).

[13] Lustig Y (2021). Neutralising capacity against Delta (B.1.617.2) and other variants of concern following Comirnaty (BNT162b2, BioNTech/Pfizer) vaccination in health care workers, Israel. Eurosurveillance.

[14] Pfizer CEO says third Covid vaccine dose likely needed within 12 months. https://www.cnbc.com/2021/04/15/pfizer-ceo-says-third-covid-vaccine-dose-likely-needed-within-12-months.html.

[15] Israel offers third shot of Pfizer COVID-19 vaccine to adults at risk | Reuters. https://www.reuters.com/world/middle-east/israel-offers-pfizer-covid-19-vaccine-booster-shots-adults-risk-2021-07-11/.

[16] Shalev V (2011). The use of an automated patient registry to manage and monitor cardiovascular conditions and related outcomes in a large health organization. Int. J. Cardiol..

[17] Chodick G, Heymann AD, Shalev V, Kookia E (2003). The epidemiology of diabetes in a large Israeli HMO. Eur. J. Epidemiol..

[18] Coresh J (2014). Decline in estimated glomerular filtration rate and subsequent risk of end-stage renal disease and mortality. JAMA.

[19] Israel Center for Disease Control. Jerusalem, I. Data from: Israel national cancer registry.

