# Supplementary material for: Correlation of SARS-CoV-2-breakthrough infections to time-from-vaccine
Source: Nat Commun. 2021 Nov 4;12:6379. doi: 10.1038/s41467-021-26672-3 (PMC8569006; doi:10.1038/s41467-021-26672-3)
Supplement: Supplementary file 1 — Supplementary Information [file 41467_2021_26672_MOESM1_ESM.pdf]

Supplementary Information

Supplementary Table 1- Unadjusted Hazard Ratio (HRs) of SARS-CoV-2 Breakthrough Infections

	N in the Early Vaccinees group	N in the Late Vaccinees group	N in each matched[1] group	N of new cases[2] Early	N of new cases[2] Late	HR[3] (95% CI)	P
Model 1: Comparing Early Vaccinees (January-February 2021) to Late Vaccinees (March-April 2021)							
All	935,781	416,663	329,177	1151	760	1.52 (1.38-1.66)	5.91E-19
16-39 yr	242,943	302,229	215,919	807	530	1.52 (1.37-1.70)	5.00E-14
40-59 yr	394,896	86,559	85,502	283	191	1.48 (1.23-1.78)	2.62E-05
≥60 yr	297,942	27,875	27,756	61	39	1.56 (1.05-2.34)	0.029
Model 2: Comparing Vaccinees by month in 2021							
Jan/Feb	475,281	460,500	233,622	1019	795	1.28 (1.17-1.41)	1.47E-07
Jan/Mar	475,281	371,929	145,431	563	351	1.61 (1.40-1.83)	3.44E-12
Jan/Apr	475,281	44,734	40,356	156	70	2.23 (1.68-2.96)	2.43E-08
Feb/Mar	460,500	371,929	272,243	929	666	1.40 (1.26-1.54)	5.20E-11
Feb/Apr	460,500	44,734	43,649	148	75	1.98 (1.50-2.61)	1.57E-06
Mar/Apr	371,929	44,734	44,402	101	76	1.33 (0.99-1.79)	0.061

Between Early and Late Vaccinee Groups
