# Supplementary material for: Correlation of SARS-CoV-2-breakthrough infections to time-from-vaccine
Source: Nat Commun. 2021 Nov 4;12:6379. doi: 10.1038/s41467-021-26672-3 (PMC8569006; doi:10.1038/s41467-021-26672-3)
Supplement: Supplementary file 3 — Reporting Summary [file 41467_2021_26672_MOESM3_ESM.pdf]

nature portfolio

Corresponding author(s): Roni Lotan

Last updated by author(s): Sep 19, 2021

Reporting Summary

Nature Portfolio wishes to improve the reproducibility of the work that we publish. This form provides structure for consistency and transparency in reporting. For further information on Nature Portfolio policies, see our Editorial Policies and the Editorial Policy Checklist.

Statistics

For all statistical analyses, confirm that the following items are present in the figure legend, table legend, main text, or Methods section.

n/a	Confirmed	
☐	☒	The exact sample size (*n*) for each experimental group/condition, given as a discrete number and unit of measurement
☐	☒	A statement on whether measurements were taken from distinct samples or whether the same sample was measured repeatedly
☐	☒	The statistical test(s) used AND whether they are one- or two-sided<br>*Only common tests should be described solely by name; describe more complex techniques in the Methods section.*
☐	☒	A description of all covariates tested
☐	☒	A description of any assumptions or corrections, such as tests of normality and adjustment for multiple comparisons
☐	☒	A full description of the statistical parameters including central tendency (e.g. means) or other basic estimates (e.g. regression coefficient) AND variation (e.g. standard deviation) or associated estimates of uncertainty (e.g. confidence intervals)
☐	☒	For null hypothesis testing, the test statistic (e.g. *F*, *t*, *r*) with confidence intervals, effect sizes, degrees of freedom and *P* value noted<br>*Give P values as exact values whenever suitable.*
☒	☐	For Bayesian analysis, information on the choice of priors and Markov chain Monte Carlo settings
☒	☐	For hierarchical and complex designs, identification of the appropriate level for tests and full reporting of outcomes
☒	☐	Estimates of effect sizes (e.g. Cohen's *d*, Pearson's *r*), indicating how they were calculated

Our web collection on statistics for biologists contains articles on many of the points above.

Software and code

Policy information about availability of computer code

Data collection	Retrospective electronic health recodes data from Maccabi Health Services
Data analysis	Analyses were performed using Python version 3.1 with the stats model package. Code is available at: https://github.com/barakm-ki/Correlation-of-SARS-CoV-2-Breakthrough-Infections-to-Time-from-vaccine

For manuscripts utilizing custom algorithms or software that are central to the research but not yet described in published literature, software must be made available to editors and reviewers. We strongly encourage code deposition in a community repository (e.g. GitHub). See the Nature Portfolio guidelines for submitting code & software for further information.

Data

Policy information about availability of data

All manuscripts must include a data availability statement. This statement should provide the following information, where applicable:

- Accession codes, unique identifiers, or web links for publicly available datasets
- A description of any restrictions on data availability
- For clinical datasets or third party data, please ensure that the statement adheres to our policy

According to the regulations of the Israel Ministry of Health, individual-level data cannot be shared openly. Specific requests for remote access to de-identified community-level data should be referred to Maccabitech, Maccabi Healthcare Services Institute for Research & Innovation.
publicly available. Due to restrictions, these data can be accessed only by request to the authors and/or Maccabi Health Services.

Field-specific reporting

Please select the one below that is the best fit for your research. If you are not sure, read the appropriate sections before making your selection.

☒ Life sciences ☐ Behavioural & social sciences ☐ Ecological, evolutionary & environmental sciences

For a reference copy of the document with all sections, see nature.com/documents/nr-reporting-summary-flat.pdf

Life sciences study design

All studies must disclose on these points even when the disclosure is negative.

Sample size	No sample size calculation was performed since sample size was determined by the number of patients in the electronic health records.
Data exclusions	Of 1,395,134 MHS members over the age of 16 who received the second dose of the vaccine between January and April of 2021, 1,352,444 were eligible for the study. 27,143 individuals did not receive the second dose according to the guidelines, and 15,547 individuals were tested positive to SARS-CoV-2 prior to the study period.
Replication	NA. This was an observational study describing the breakthrough infection rates using electronic health records.
Randomization	Randomization was not applicable in this study since we used existing retrospective electronic health records.
Blinding	Blinding was not applicable in this study since we analyzed existing anonymized retrospective electronic health records.

Reporting for specific materials, systems and methods

We require information from authors about some types of materials, experimental systems and methods used in many studies. Here, indicate whether each material, system or method listed is relevant to your study. If you are not sure if a list item applies to your research, read the appropriate section before selecting a response.

Materials & experimental systems

n/a	Involved in the study	
☒	☐	Antibodies
☒	☐	Eukaryotic cell lines
☒	☐	Palaeontology and archaeology
☒	☐	Animals and other organisms
☐	☒	Human research participants
☒	☐	Clinical data
☒	☐	Dual use research of concern

Methods

n/a	Involved in the study	
☒	☐	ChIP-seq
☒	☐	Flow cytometry
☒	☐	MRI-based neuroimaging

Human research participants

Policy information about studies involving human research participants

Population characteristics	The study population consisted of all MHS members aged 16 and above who received the second dose of the vaccine between January and April 2021. Individuals were considered fully vaccinated if they received two doses of the BNT162b2, the second one administered within the 21-to-28-day interval set by national guidelines. Of 1,395,134 MHS members over the age of 16 who received the second dose of the vaccine between January and April of 2021, 1,352,444 were eligible for the study. 27,143 individuals did not receive the second dose according to the guidelines, and 15,547 individuals were tested positive to SARS-CoV-2 prior to the study period.
Recruitment	No recruitment.
Ethics oversight	The study protocol was approved by Maccabi Health Services' institutional review board (MHS-0100-20).

Note that full information on the approval of the study protocol must also be provided in the manuscript.
